# Radiomics Model Based on MR Images to Discriminate Pancreatic Ductal Adenocarcinoma and Mass-Forming Chronic Pancreatitis Lesions

**DOI:** 10.3389/fonc.2021.620981

**Published:** 2021-03-24

**Authors:** Yan Deng, Bing Ming, Ting Zhou, Jia-long Wu, Yong Chen, Pei Liu, Ju Zhang, Shi-yong Zhang, Tian-wu Chen, Xiao-Ming Zhang

**Affiliations:** ^1^ Sichuan Key Laboratory of Medical Imaging and Department of Radiology, Affiliated Hospital of North Sichuan Medical College, Nanchong, China; ^2^ Department of Radiology, Deyang People’s Hospital, Deyang, China; ^3^ Department of Radiology, Ruijin Hospital, Shanghai Jiao Tong University School of Medicine, Shanghai, China

**Keywords:** radiomics, magnetic resonance imaging, pancreatic ductal adenocarcinoma, mass-forming chronic pancreatitis, machine learning

## Abstract

**Background:**

It is difficult to identify pancreatic ductal adenocarcinoma (PDAC) and mass-forming chronic pancreatitis (MFCP) lesions through conventional CT or MR examination. As an innovative image analysis method, radiomics may possess potential clinical value in identifying PDAC and MFCP. To develop and validate radiomics models derived from multiparametric MRI to distinguish pancreatic ductal adenocarcinoma (PDAC) and mass-forming chronic pancreatitis (MFCP) lesions.

**Methods:**

This retrospective study included 119 patients from two independent institutions. Patients from one institution were used as the training cohort (51 patients with PDAC and 13 patients with MFCP), and patients from the other institution were used as the testing cohort (45 patients with PDAC and 10 patients with MFCP). All the patients had pathologically confirmed results, and preoperative MRI was performed. Four feature sets were extracted from T1-weighted imaging (T1WI), T2-weighted imaging (T2WI), and the artery (A) and portal (P) phases of dynamic contrast-enhanced MRI, and the corresponding radiomics models were established. Several clinical characteristics were used to discriminate PDAC and MFCP lesions, and clinical model was established. The results of radiologists’ evaluation were compared with pathology and radiomics models. Univariate analysis and the least absolute shrinkage and selection operator algorithm were performed for feature selection, and a support vector machine was used for classification. The receiver operating characteristic (ROC) curve was applied to assess the model discrimination.

**Results:**

The areas under the ROC curves (AUCs) for the T1WI, T2WI, A and, P and clinical models were 0.893, 0.911, 0.958, 0.997 and 0.516 in the primary cohort, and 0.882, 0.902, 0.920, 0.962 and 0.649 in the validation cohort, respectively. All radiomics models performed better than clinical model and radiologists’ evaluation both in the training and testing cohorts by comparing the AUC of various models, all P<0.050. Good calibration was achieved.

**Conclusions:**

The radiomics models based on multiparametric MRI have the potential ability to classify PDAC and MFCP lesions.

## Background

Pancreatic ductal adenocarcinoma (PDAC) is a malignancy with an overall 5-year survival rate of less than 10% and is a third-leading cause of death among cancers (1). Radical resection is the only possible curative treatment in patients with PDAC (2). However, radical resection may lead to unnecessary complications and risk of death in benign lesions (3). Mass-forming chronic pancreatitis (MFCP) is condition causing benign lesions to form in the pancreas; these lesions easily mimic PDAC lesions. In clinical practice, MFCP is difficult to differentiate from PDAC due to their similar presentations of abdominal pain, weight loss, pancreatic insufficiency, and overlapping radiologic features (4–7). Overall, 5-11% of patients who underwent a pancreaticoduodenectomy did so because what were considered pancreatic malignancies turned out to be benign lesions of the pancreas (3).Therefore, it is important to discriminate PDAC lesions from MFCP lesions because their prognosis and management are so different (8–10). Although many diagnostic methods for differentiating PDAC lesions from MFCP lesions have been developed (6, 7, 11–13). Differentiation remains the most challenging issue faced by radiologists because of the substantial overlap in imaging findings (14, 15). For example, PDAC and MFCP lesions result in the dilation of the main pancreatic duct and the common bile duct (16). Both PDAC and MFCP lesions are composed of dense fibrous tissue (12). Biopsy is the most reliable diagnostic method for distinguishing PDAC and MFCP lesions. However, there are certain limitations, such as a significant false negative rate and many complications, the negative rate ranges from 46%-80% (17).

Radiomics can noninvasively extract a large number of features invisible to the naked eye from traditional pictures and translate them into high-dimensional data through machine learning methods (18). Based on the nature of texture analysis, radiomics relies on the objective calculation by a computer rather than the subjective diagnosis of radiologist (19). The utility of radiomics has been applied to the discrimination of lesions in the lungs, brain, and breasts (20–22). No study has reported the utility of radiomic-based model in the discrimination of PDAC and MFCP lesions. Compared with CT examinations, a series of previous studies have demonstrated that MRI has a better diagnostic performance for differentiating MFCP lesions from PDAC lesions (12, 23).

The aim of our study was to develop and validate radiomics models that extract features from multiparametric MRI and clinical features to differentiate PDAC lesions from MFCP lesions.

## Methods

### Patient Selection

**Figure 1:**
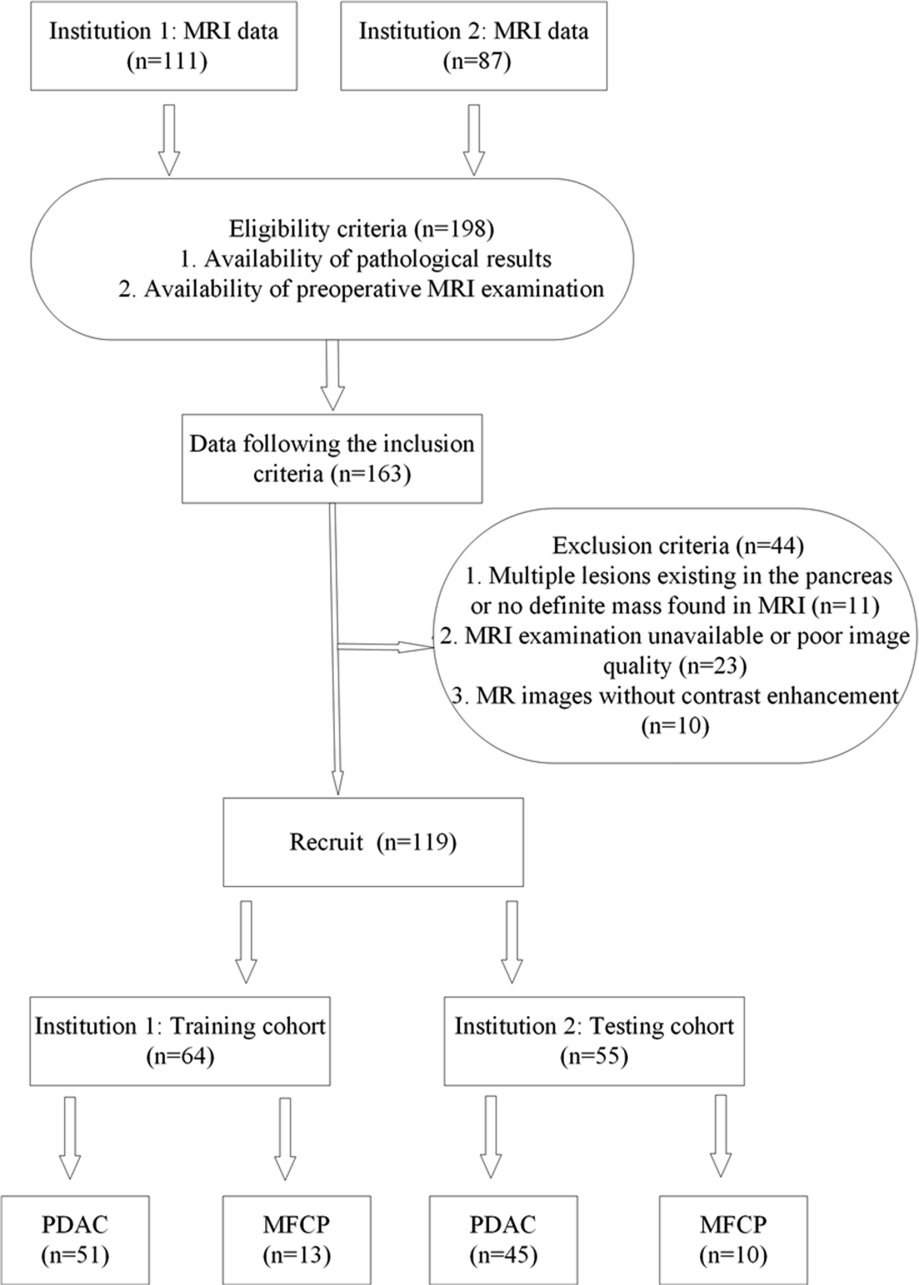
The flowchart of patient enrollment in our study. MRI, magnetic resonance imaging; PDAC, pancreatic ductal adenocarcinoma; MFCP, mass-forming chronic pancreatitis.

Our study was conducted at two institutions in SiChuan province. Ethical approval to perform this retrospective study was obtained from each Institutional Review Board (IRB), and informed consent was waived. Patients with a pathological diagnosis of either PDAC or MFCP from March 2016 to June 2019 were identified by searching the pathology database from the two centers. MFCP is defined as chronic inflammation with focal mass formation confirmed (24). In total, 198 consecutive patients were identified. Patients were included in our study if they met the following criteria: (1) patients had a definitive diagnosis of PDAC or MFCP confirmed by histopathology; (2) patients underwent an upper-abdomen MRI examination before surgery or biopsy. We used the following exclusion criteria: (1) multiple lesions in the pancreas or no definite mass were found with MRI; (2) MRI examination was not performed at our institutions or the image quality was poor; and (3) MRI was performed without contrast enhancement. The final study sample included 119 patients (**Figure 1**). The dataset from institution 1 was used as the training cohort, and the dataset from institution 2 constituted the testing cohort. Some clinic and imaging characteristics were included, such as sex, age, lesion location, lesion size, the diameter of the largest cross section of the main pancreatic duct (MPD), the diameter of the largest cross section of the common bile duct (CBD) and the presence status of CA19-9. We defined the CA19-9 result as normal at 0 to 34 U/ml, and abnormal when exceeding.

### MRI Image Examination

All patients underwent a 3.0-T MR examination (MR 750, GE Medical Systems, Waukesha, WI, USA, and Achieva, Philips, the Netherlands). The general sequences consisted of T2-weighted imaging (T2WI), precontrast T1-weighted imaging (T1WI), and the arterial phase and portal-venous phase of dynamic contrast-enhanced MRI (DCE-MRI). For the DCE-MRI sequence, 20 mL of gadopentetate dimeglumine (Magnevist; Schering, Guangzhou, China) was administered intravenously with a pressure injector (Spectris MR Injection System, MEDRAD, Inc., USA) at 2–3 mL/s, followed by a 20-mL saline solution flush. The scanning times were set to 30 s and 60 s after the contrast agent was injected to obtain the arterial phase and portal-venous phase images, respectively. Detailed information on the acquisition parameters is described in **Table 1**.

**Table 1:** The parameters of the 3.0-T MRI scanners.

	TR	TE	Flip angle	Selection thickness (mm)	Matrix	FOV
GE-MR750 T1WI	4.2	2.6	15	5	384×224	26×33
Achieva T1WI	4	2	10	4	160×160	246×320
GE-MR750 T2WI	2500	100	90	5	320×256	39×33
Achieva T2WI	1200	80	90	7	208×186	261×335
GE-MR750 DCE-MRI	4.2	2.6	15	5	384×224	26×33
Achieva DCE-MRI	4	2	10	4	160×160	246×320

### Image Interpretation

**Figure 2:**
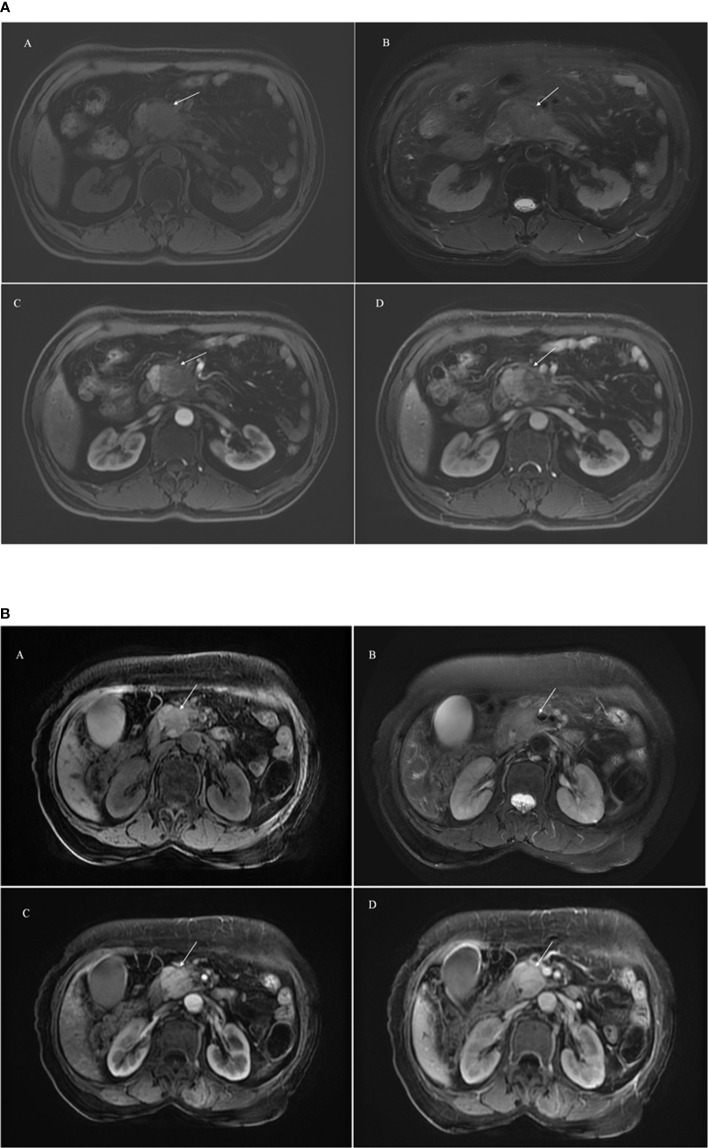
A 65-year male patient with PDAC showing an ill-defined mass (arrowhead) in the head of pancreas (2a). A hypointensity mass shows on axial T1WI **(A)**, hyperintensity on axial T2WI **(B)**, and unobvious enhancement during the artery **(C)** and portal venous **(D)** phase. A 58-year male patient with MFCP showing an mass (arrowhead) in the head of pancreas (2b). A hypointensity mass shows on axial T1WI **(A)**, hyperintensity on axial T2WI **(B)**, and gradual enhancement during the artery **(C)** and portal venous **(D)** phase.

Two experienced radiologists (with 4 and 8 years of experience in abdominal imaging) processed all the MR images independently and were blinded to the pathological results. They calculated the size of each lesion by measuring the long and short diameters of the largest cross-section of the lesion, measured the diameter of maximal cross-section of MPD and CBD. PDAC was defined an irregular mass was hypointensity on axial T1WI, hyperintensity on axial T2WI, and unobvious enhancement during the artery, portal venous and delayed phases. MFCP was defined a clear boundary mass was hypointensity on axial T1WI, hyperintensity on axial T2WI, and gradual enhancement during the dynamic enhancement (**Figure 2**). When their results were inconsistent, the final decision was determined as PDAC or MFCP after discussion. We compared the results of radiologist’s evaluation with pathological results and calculated the discrimination such as accuracy, sensitivity and so on.

### Image Segmentation, Preprocessing and Feature Extraction

**Figure 3:**
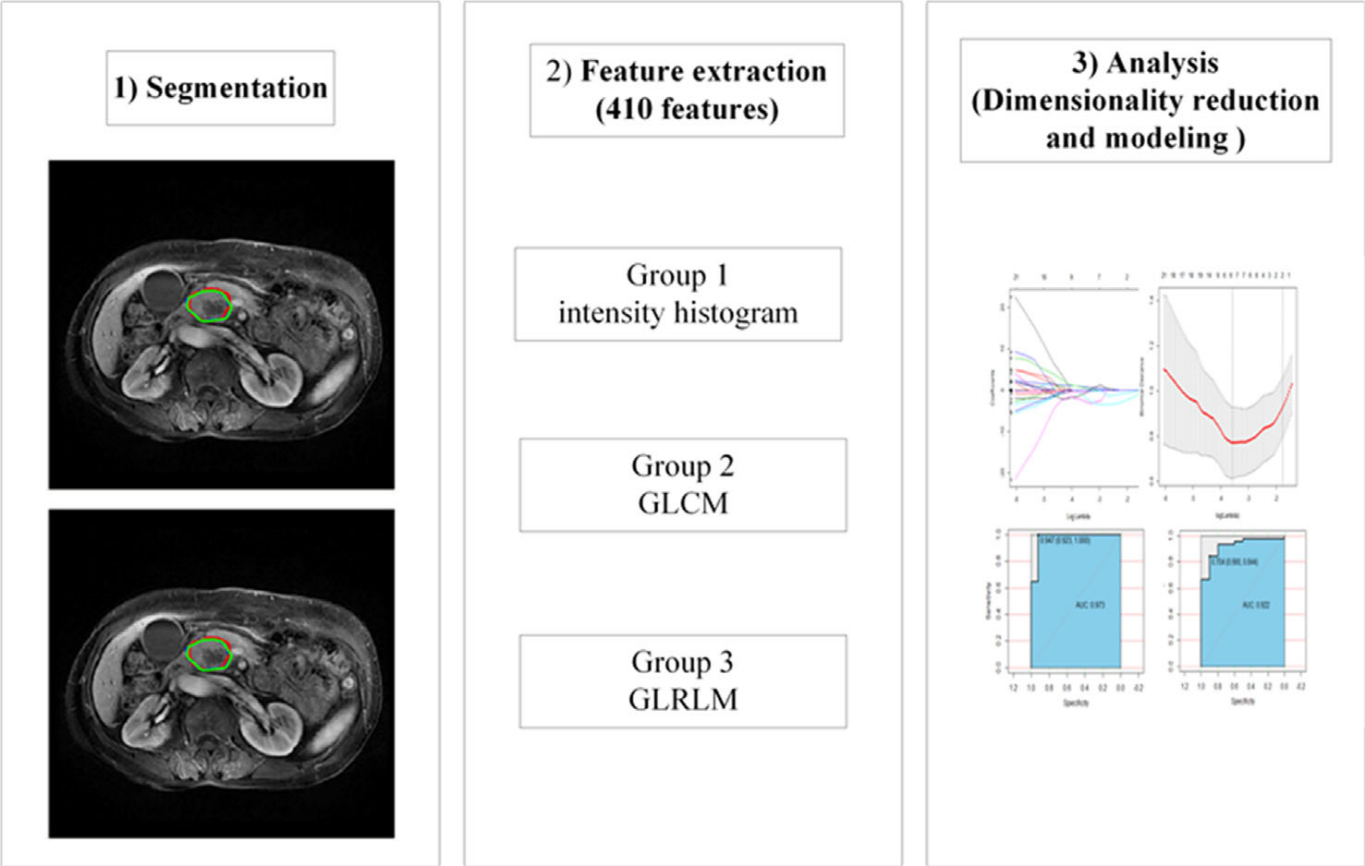
The workflow of radiomic. GLCM, gray level co-occurrence matrix; GLRLM, gray-level run-length matrix.

Two experienced radiologists manually delineated the region of interest (ROI) based on T1WI, T2WI, the arterial phase (A) and portal-venous phase (P) on the basis of the largest size of the tumors in an axial image slice, corresponding four independent feature sets generated and radiomics models established. To eliminate the volumetric effect of the peripancreatic fat space or normal pancreas, they slightly delineated within the lesion (25). The process was implemented by using IBEX (β1.0, http://bit.ly//IBEXMDAnderson), an open source software program running on MATLAB 2016b (The MathWorks Inc), the workflow of radiomic showed in **Figure 3**. before extracted features, A Laplacian-of-Gaussian (LoG) filter was applied for imaging preprocessing, which will help improve the efficiency of capturing phenotypic features associated with tumor heterogeneity (26). before feature extraction, all MRI sequences were normalized using Z-scores in order to reduce the potential impact introduced by scanning parameters, protocols, scanners, vendors and eliminate the influence of dimensions (27), was performed in the training and validation data sets to improve the repeatability of the analysis (See **Supplemental Material 1**, which elucidated the preprocessing methods for the image and data).

Then, we selected three group features extracted from IBEX: the intensity histogram using the common fundamental measurement value to reflect the distribution of gray pixels in the image; the gray-level co-occurrence matrix (GLCM), which it reflects the measurements of the texture image by pixels with the same gray level and is mainly used for linear texture analysis; and the gray-level run-length matrix (GLRLM), which reflects the comprehensive information about the change in gray levels in terms of the step size and direction, and reflects the arrangement rules about different pixels. In total, 410 radiomics features were identified in each independent feature set (See **Supplementary Table 1**).

### Intraobserver and Interobserver Agreement

Fifty patients were randomly selected to have the outline of the ROI from T1WI, T2WI, the artery and portal venous phase of multisequence MRI drawn, and the corresponding feature subsets were generated to evaluate the reproducibility of radiomics. To assess the intraobserver agreement, observer 1 delineated the ROI twice, with a time between delineations of more than one week. To evaluate the interobserver agreement, observer 2 independently delineated the ROI once and compared these results with the first results from observer 1. The intra- and interobserver agreement were assessed through intraclass correlation coefficients (ICC), and an ICC score greater than 0.75 indicates a good agreement (28). Not all radiomics features achieved satisfactory conformance due to the nature of the voxel size and gray-level dependence (29). Observer 1 completed all the image segmentations.

### Dimensionality Reduction and Radiomics Feature Selection

Dimensionality reduction methods were applied to the training group to avoid dimensional disasters and reduce deviations from the radiomics features. First, independent samples t-test or Mann-Whiney U tests were performed to identify features in each feature set that were significantly different between PDAC lesions and MFCP lesions. To reduce the risk of type I error, a false discovery rate (FDR) was applied to correct the P values. Then, the least absolute shrinkage and selection operator (LASSO) algorithm was performed for dimensionality reduction and feature selection before classification (30). One standard error (1-SE standard, a simple model) was used to adjust the regularization parameters (λ) and feature selection using 10-fold cross-validation.

### Development and Validation of the Optimal Radiomics Signature

Radiomics features of each feature subset were selected by the above procedure, and a support vector machine (SVM) with a Gaussian kernel was applied to establish a nonlinear radiomics model. The kernel’s parameter size (γ, gamma,∈ [0.001,1]) and the regularization parameters (C, cost,∈[1,100])) were optimized, and 10-fold cross-validation of the SVM kernel function was performed to select the best-performing models. Four radiomics models were established. An independent clinical model was established using classical imaging and clinical factors, for example, the size of lesion, the diameter of the largest cross section of the MPD and CBD, the status of CA19-9 followed the same tuning procedure described in the development of radiomics models. The performance of the four radiomics and clinical models was calculated by the area under the receiver operating characteristic (ROC) curve (AUC) and other evaluation metrics, such as the sensitivity, and specificity. The radiomics and clinical models were also assessed in the testing cohort. DeLong test was implemented to compare the AUC of four radiomics models, clinical model and radiologists’ evaluation both in the training and testing cohort.

### Statistical Analysis

Regarding the clinical data, continuous variables, including the age of the patient and size of the lesion were assessed with independent samples t-test or Mann-Whiney U tests based on their distributions. Categorical variables, including the sex of the patient, the result of CA19-9 and the location of the lesion, were assessed with Pearson chi-square test or Fisher exact test. To assess the radiologist’s evaluation and pathological results, Pearson Chi-square was performed. These data were analyzed by Statistical Package for the Social Sciences (SPSS; IBM SPSS Statistics for Windows, Version 23.0, IBM Corp, Armonk, NY, USA). The dimensionality reduction and model building processes of the radiomics features, including the intensity histogram, GLCM and GLRLM of each model, were implemented in R (Version 3.5.2, https://www.r-project.org/). The LASSO regression, SVM model and ROC curve analyses were performed by means of the “*ggplot2*”, “*e1071*” and “*pROC*” packages, respectively. In all tests of differences, a P-value less than 0.050 was considered a statistical significant.

## Results

### Clinical Data

In total, 119 patients from two independent institutions were included in the study. When comparing the results between radiologists’ evaluation and pathology, no significant difference was existed (χ^2^ = 0.152, P=0.076). Radiologists had better accuracy (80.7%), positive predictive value (92.7%) and sensitivity (84.8%) in identifying PDAC and MFCP, however, the specificity (50%) and negative predictive value (30.4%) were poor. The composition of PDAC was higher than that of MFCP in the training and testing cohorts, but no statistically significant differences existed between the two cohorts (P=0.769, χ2 = 0.086). The baseline characteristics of the two cohorts are recorded in **Table 2**. In both the primary and testing cohorts, there were no significant differences between patients with PDAC and MFCP in terms of age (P=0.707 vs 0.526), sex (P=0.507 vs 0.303), lesion location (P=0.648 vs 0.615), lesion size (P=0.081 vs 0.441), the diameter of the largest cross section of the MPD and CBD (P=0.745 vs 0.07 and P=0.761 vs 0.142). The CA19-9 level was significantly different in the primary cohort (P<0.050) while no significant difference in the testing cohort (P=0.051).

**Table 2:** Patient characteristics and MR image findings for the primary and validation cohorts.

	The primary cohort		P value	The validation cohort		P value
	PDAC (n = 51)	MFCP (n = 13)		PDAC (n = 45)	MFCP (n = 10)	
Age (years)	63 (52, 68)	60 (53, 66)	0.707	62 (54, 69)	57 (52,71)	0.526
Sex			0.507			0.303
Male	37	10		22	7	
Female	14	3		23	3	
Location			0.648			0.615
Head or neck	46	12		39	9	
Body or tail	5	1		6	1	
Size (cm^2^)	5.75 (4.14, 9.99)	5.32 (2.62, 6.09)	0.081	6.96 (4.39,9.445)	6.60 (4.28,7.20)	0.441
MPD (cm)	0.46 (0.32, 0.63)	0.45 (0.29, 0.78)	0.745	0.54 (0.35, 0.74)	0.33 (0.28, 0.44)	0.07
CBD (cm)	1.30 (0.70, 1.60)	1.50 (0.75, 1.85)	0.761	1.30 (0.50, 1.65)	1.00 (0.48, 1.30)	0.142
CA19-9	9	10	<0.05*	12	7	0.051
Normal	42	3		33	3	
High						

*represents a statistically significant difference.

MPD: the diameter of the largest cross section of the main pancreatic duct (MPD); CBD: the diameter of the largest cross section of the common bile duct (CBD).

### Intraobserver and Interobserver Agreement

Regarding the interobserver agreement of radiomics features, the mean values were 0.942 (range: 0.428 to 0.998), 0.943 (range: 0.269 to 0.994), 0.961 (range: 0.505 to 0.999), 0.955 (range: 0.199 to 0.999) for the T1WI, T2WI, A and P feature subsets, respectively. For the intraobserver agreement, the mean values were 0.934 (range: 0.442 to 0.999), 0.943 (range: 0.269 to 0.994), 0.940 (range: 0.378 to 0.999), 0.955 (range: 0.199 to 0.999) for the T1WI, T2WI, A and P feature subsets, respectively. Ultimately, the number of excluded features in the T1WI, T2WI, A, and P feature subsets were 27, 21, 11 and 13, respectively, and the remaining features were analyzed in the next section.

### Dimensionality Reduction and Radiomics Feature Selection

The number of selected features of each separate subset after univariate analysis and LASSO algorithm implementation are shown in **Table 3**. The features selected in each feature set were used in further modeling analysis. Dimensionality reduction was performed through two steps; there were 5, 7, 7, and 9 features included in T1WI, T2WI, A and P models, respectively for models building (See **Supplemental Material 2**, which elucidate the information for the selected features).

**Table 3:** The numbers of features selected through the intraobserver and interobserver agreement tests, univariate analysis and the LASSO algorithm.

	T1WI	T2WI	A	P
Original features	410	410	410	410
ICC analysis	383	389	399	397
Univariate analysis and FDR correct	222	287	228	2218
LASSO	5	7	7	9

### Development and Validation of the Optimal Radiomics Signature

**Figure 4:**
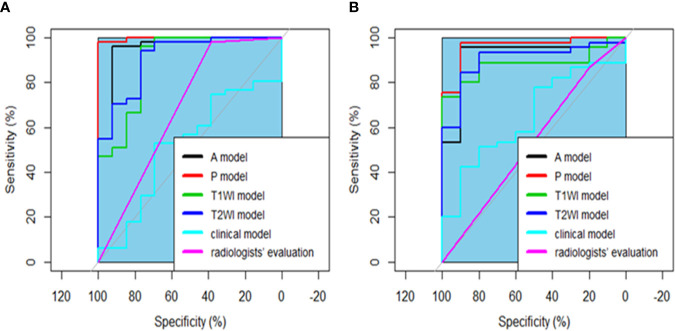
The ROC curve for the four radiomics models, clinical model and radiologists’ evaluation for the training **(A)** and testing **(B)** cohorts. There was no significant difference among the four radiomics models by comparing the AUC of different models (all P > 0.050). All radiomics performed better than clinical model and radiologists’ evaluation by comparing the AUC of various models, all P < 0.050.

The four radiomics models achieved good performance in the training and testing cohorts based on SVM modeling. The AUC of T1WI model, T2WI model, A model and P model were 0.893 [95% confidence interval (CI): 0.780-1], 0.911 (95%CI: 0.823-0.999), 0.958 (95%CI: 0.889-1), 0.997(95%CI: 0.990-1) in the training cohorts, The AUC of T1WI model, T2WI model, A model and P model were 0.882 (95% CI: 0.792-0.972), 0.902 (95%CI: 0.809-0.995), 0.920 (95%CI: 0.821-1), 0.962 (95%CI: 0.907-1) in the testing cohorts. The AUC of clinical model were 0.516 and 0.649 in the training and testing cohorts. The detailed results were shown in **Table 4**. When comparing the AUC across the four radiomics models *via* the DeLong test, no significant differences existed between pairs of models (all P > 0.05). Interestingly, the performance of radiomics models were superior to clinical model and radiologists’ evaluation (P<0.050). The ROC curves of five models and radiologists’ evaluation in the primary and validation models are shown in **Figure 4**.

**Table 4:** The performance of the radiomics and clinical models using support vector machine method in the training and testing cohorts.

		Sensitivity	Specificity	AUC (95% CI)
T1WI Model	Training cohort	0.961	0.769	0.893 (0.780-1)
	Testing cohort	1	0.733	0.882 (0.792-0.972)
T2WI Model	Training cohort	0.941	0.769	0.911 (0.823-0.999)
	Testing cohort	0.844	0.900	0.902 (0.809-0.995)
A Model	Training cohort	0.961	0.923	0.958 (0.889-1)
	Testing cohort	0.956	0.900	0.920 (0.821-1)
P Model	Training cohort	0.980	1	0.997 (0.990-1)
	Testing cohort	0.978	0.900	0.962 (0.907-1)
Clinical Model	Training cohort	0.529	0.692	0.516 (0.340-0.692)
	Testing cohort	0.422	0.900	0.649 (0.474-0.823)

## Discussion

In our study, we compared the radiologists’ evaluation and pathological results of PDAC and MFCP, there was no significant difference (P=0.076). Radiologists had a potential ability to assess the lesions. However, the specificity and negative predication value was poor. It may lead to overtreatment of MFCP, which was consistent with previous study (3). We constructed clinical model by combining the size of lesion, the diameter of the largest cross section of the MPD and CBD to identify PDAC and MFCP, however, no good performance was achieved. These results showed it was difficult to make a diagnosis of the two diseases based on the traditional image and clinical data. Interestingly, we develop and validate a multiparameter MRI-based radiomics method for noninvasive differentiation of PDAC from MFCP lesions before surgery, and achieved good performance in both the training and testing cohorts. Every single feature subset extracted from T1WI, T2WI, the artery and portal venous phase performed well. These results suggest that our radiomics model can be used as a quantitative tool to discriminate PDAC from MFCP lesions preoperatively. While this discovery was an encouraging initial step, it is necessary to focus more on a better understanding of the basic biological principles of measurement through radiomic analysis and how to better integrate it with other analytical methods for better clinical application.

Some clinical and imaging characteristics were included in this study. Only the serum CA19-9 level was significantly different between patients with PDAC and MFCP in the training cohort, there was no significant difference in the validation cohort. this may be related with the small number of patients in the validation cohort. It indicates that CA19-9 may be regarded as a serum biomarker to identified PDAC from MFCP, however, serum CA19-9 had a high false negative rate. Ren et al. (31) demonstrated that no statistically significant difference with respect to degree of pancreatic ductal dilatation was observed between PDAC and MFCP. Our conclusion is consistent with theirs. Sandrasegaran et al. (24) demonstrated that there was no significant difference in the lesion size between PDAC and MFCP. The AUC for size of mass and pancreatic duct dilatation in differentiating malignant and benign entities are 0.697 and 0.589-0.622, their results suggested that the clinical and imaging features is poor in differentiate MFCP from PDAC.

In our study, all the A model, P model, T1WI model and T2WI model achieved good performance in the training and testing cohorts(the AUCs were 0.893, 0.911, 0.958 and 0.997 vs 0.882, 0.902, 0.920 and 0.962), The reason for these findings may be associated with the fact that fat-suppressed T1WI, T2WI and dynamic contrast-enhanced MRI had a good diagnostic effect in detecting pancreatic cancer, and the tumor-pancreas contrast was best 40-70 s after injection of the contrast agent (32, 33).

Previous studies have developed several methods for discriminating PDAC from MFCP lesions. Previous studies applied perfusion CT to distinguish PDAC from MFCP lesions, and some perfusion parameters, such as blood flow and blood volume (6, 7), could provide information to identify PDAC and MFCP lesions. However, this method involves high amounts of radiation, and the iodide ion contrast agent is not suitable for patients with renal dysfunction. Ren et al. (31) showed that CT texture analysis demonstrates great potential to differentiate MFCP from PDAC. The combined model based on imaging features and texture features reveal high pooled sensitivity of 94%, specificity of 92%. Some previous studies had demonstrated that MRI is superior to other preoperative imaging techniques in identifying in the diagnosis of MFCP and PDAC. Several studies applied MRI to identify MFCP from PDAC. Some studies suggested that diffusion weighted imaging (DWI) can reflect the differences between PDAC and MFCP lesions; a meta-analysis (13) combined several DWI-related studies and the summary AUC of 0.91. Our study was superior to previous results and robust. Shi et al. (11) used MR elastography to differentiate PDAC and MFCP lesions, and the mass stiffness and stiffness ratio achieved AUCs of 0.882 and 0.955, respectively. Our study developed and validated radiomics models to classify PDAC and MFCP lesions based on multisequence MRI, and these results were consistent with this finding. In addition, the MR elastography technique has a lower resolution than routine MRI.

Radiomics is noninvasive, inexpensive and robust. The high-dimensional imaging features of radiomics provide more detailed information on tumors that are difficult to detect with the naked eye. Our radiomics models achieved good performance; in the training cohorts, the AUC, sensitivity, and specificity performed well in the T1WI, T2WI, A and P models. The discriminative performance of the radiomics model was also remarkable in the validation cohorts.

Some limitations exist in our research. Firstly, the sample size was small. In addition, the composition of the PDAC and MFCP samples are very different; however, there was no difference in the composition ratio between the training and testing cohorts. Multicenter and large-scale study would need to be performed. Last, we performed a two-dimensional analysis of the area of interest for the largest section of the lesion rather than a three-dimensional analysis of the entire lesion volume. This approach is less labor intensive but less sensitive to intratumor variations. however, previous study (34) identified PDAC and autoimmune pancreatitis using two-dimensional analysis and achieved good performance, and our results were satisfactory.

## Conclusion

In conclusion, our results show that radiomic models based on multiparametric MRI have the potential to distinguish PDAC lesions from MFCP lesions. This method needs to be validated in a larger sample size for better clinical application.

## Data Availability Statement

The original contributions presented in the study are included in the article/**Supplementary Material**. Further inquiries can be directed to the corresponding author.

## Ethics Statement

The studies involving human participants were reviewed and approved by Ethics Committee of Affiliated Hospital of North Sichuan Medical College and Deyang People’s Hospital. The ethics committee waived the requirement of written informed consent for participation.

## Author Contributions

X-MZ designed the research study. TZ, JZ, T-WC, and YC conducted the study and collected the data. S-YZ, PL, and JLW analyzed the data. YD and BM performed the segmentation of image, processing data, and writing paper. All authors contributed to the article and approved the submitted version.

## Funding

The Major frontier program of applied basic research in Sichuan province, No. 2018JY0012.

## Conflict of Interest

The authors declare that the research was conducted in the absence of any commercial or financial relationships that could be construed as a potential conflict of interest.
