# Supplementary material for: Radiomics Model Based on MR Images to Discriminate Pancreatic Ductal Adenocarcinoma and Mass-Forming Chronic Pancreatitis Lesions
Source: Front Oncol. 2021 Mar 24;11:620981. doi: 10.3389/fonc.2021.620981 (PMC8025779; doi:10.3389/fonc.2021.620981)
Supplement: Supplementary file 1 [file DataSheet_1.docx]

**Supplementary material**

**Supplementary material 1**

**S1. Feature extraction**

Three groups of radiomic features were extracted from IBEX: 1) the intensity histogram; 2) the gray-level co-occurrence matrix (GLCM); and 3) the gray-level run-length matrix (GLRLM). Feature explanation was supplemented in IBEX (β1.0, <http://bit.ly/IBEX_MDAnderson>, the University of Texas MD Anderson Cancer Center, Houston, USA). For the percentiles and percentile area of the intensity histogram, the calculation range is 5th percentile ~ 95th percentile (5% intervals). The 25th, 50th, 75th, and 95th quantiles were calculated; the remaining 6 features were calculated by one parameter. For the GLCM, five directions (*θ* = 0°, 45°, 90°, 135° and 333° plus in IBEX) and three offsets (*d*= 1, 4, 7) were measured for a total of 330 generated features. For the GLRLM, three directions (*θ* = 0°, 90°, 333° plus in IBEX) and one offset (*d*= 1) were measured for a total of 33 generated features. In total, 410 features were extracted from each sequence for each patient. All extracted features are presented in Supplementary Table 1.

**S2.** **Preprocessing methods for the image and data**

S2.1 Z-score standardization

Different radiomics features have different ranges of values, and features of different magnitudes are difficult to compare. Before further analysis, we used z-score standardization to eliminate the effects of different dimensions by scaling the values to a mean of 0 and a standard deviation of 1 using the following formula:

z = $\frac{\chi-\mu}{\sigma}$

where *μ* is the population [mean](https://en.wikipedia.org/wiki/Mean) and *σ* is the population [standard deviation](https://en.wikipedia.org/wiki/Standard_deviation).

S2.2 Laplacian-of-Gaussian (LoG) filter

Our study was conducted at two institutions. A Laplacian-of-Gaussian (LoG) filter, which can smooth images with different parameter scale settings to reduce the effects of noise and enhance texture details, was applied for imaging preprocessing, which will help improve the efficiency of capturing phenotypic features associated with tumor heterogeneity. The value of sigma in LoG filter was 0.5.

**Supplementary material 2**

**Features for modeling**

After LASSO algorithm, in the A model, these features about the X0.1InformationMeasureCorr1, X135.7InformationMeasureCorr2, X90LongRunLowGrayLevelEmpha, X.333ShortRunEmphasis, X0ShortRunEmphasis, X90ShortRunEmphasis and Range were included. In the P model, features about X90.4InformationMeasureCorr2, X90.4ClusterProminence, X.333.1Correlation, X135.7InformationMeasureCorr1, X45.7InformationMeasureCorr2, X90.1InformationMeasureCorr2, X0.4InverseDiffMomentNorm, X0.4MaxProbability and Range were included. In the T1WI model, features about X.333.1InformationMeasureCorr2, X45.1InformationMeasureCorr2, X10PercentileArea, X90LowGrayLevelRunEmpha and X90.1Correlation were included. In the T2WI model, features about X135.4Correlation, X45.7Energy, X135.4Energy, X0.4MaxProbability, X45.1MaxProbability, X0LowGrayLevelRunEmpha and MedianAbsoluteDeviation were included. The remaining features were excluded.

**Supplementary Table 1:** The radiomics features of each patient.

| Feature classification | Feature number | Feature description |
| --- | --- | --- |
| Gray-Level Co-occurrence Matrix (GLCM) | N1-N330 | Five directions (*θ* = 0°, 45°, 90°, 135° and 333° plus in IBEX) and three offsets (*d*= 1, 4, 7) were measured for a total of 330 generated features, details are as follows: Autocorrelation, ClusterProminence, ClusterShade, ClusterTendendcy, contrast, correlation, DifferenceEntropy, Dissimilarity, Energy, Entropy, Homogeneity 1, Homogeneity 2, InformationMeasureCorr1, InformationMeasureCorr2, InverseDiffMomen1, InverseDiffMomen2, InverseVariance, Max Probability, Sum Average, Sum Entropy, Sum Variance, Variance. |
| Gray-Level Run-Length matrix (GLRLM) | N331-N363 | For the GLRLM, three directions (*θ* = 0°, 90°, 333° plus in IBEX) and one offset (*d*= 1) were measured for a total of 33 generated features, details are as follows: Gray Level Nonuniformity (GLN), High Gray Level Run Empha (HGLRE), Long Run Emphasis (LRE)，Long Run High Gray Level Empha (LRHGE), Long Run Low Gray Level Empha (LRLGLE), Low Gray Level Run Empha (LGLRE), Run Length Nonuniformity (RLN), Run Percentage (RP), Short Run Emphasis (SRE), Short Run High Gray Level Empha (SRHGLE), Short Run Low Gray Level Empha (SRLGLE). |
| Intensity Histogram | N364-N410 | For the percentiles and percentile area, the calculation range is 5th percentile ~ 95th percentile (5% intervals). The 25th, 50th, 75th, and 95th quantiles were calculated; the remaining 6 features were calculated by one parameter, details are as follows: Interquartile Range, Kurtosis, Mean Absolute Deviation, Median Absolute Deviation, Percentile, Percentile Area, Quantile, Range, Skewness |
